# Remdesivir analog as SARS-CoV-2 polymerase inhibitor: virtual screening of a database generated by scaffold replacement

**DOI:** 10.1039/d2ra00486k

**Published:** 2022-08-11

**Authors:** Mohamed A. Said, Amgad Albohy, Mohamed A. Abdelrahman, Hany S. Ibarhim

**Affiliations:** Department of Pharmaceutical Chemistry, Faculty of Pharmacy, Egyptian Russian University Badr City Cairo P.O. Box 11829 Egypt Mohamed-adel@eru.edu.eg hany-s-ibrahim@eru.edu.eg; Department of Pharmaceutical Chemistry, Faculty of Pharmacy, The British University in Egypt (BUE) Suez Desert Road El-Sherouk City Cairo 11837 Egypt; Department of Medicinal Chemistry, Institute of Pharmacy, Martin-Luther-University of Halle-Wittenberg 06120 Halle (Saale) Germany

## Abstract

By the end of 2019, a novel strain of the corona viral family named SARS-CoV-2 emerged in Wuhan, China and started to spread worldwide causing one of the most dangerous lethal pandemics. Researchers utilized various reported inhibitors and drug databases for virtual screening analysis against this novel strain. Later on, they succeeded to fish and repurpose remdesivir, an antiviral nucleotide analogue that inhibits RNA polymerase of the Ebola virus, as a promising candidate against SARS-CoV-2. In this study, we used the interactions of the co-crystallized metabolite of remdesivir with SARS-CoV-2 RdRp isozyme (PDB 7BV2) to design an analog with potential extra activity. This design was based on a scaffold replacement of a pyrrolotriazine moiety. This design was guided by a generated structure-based pharmacophore. The database generated from scaffold replacement was subjected to molecular docking and molecular dynamics simulations within the active site of SARS-CoV-2 RdRp (PDB 7BV2) to suggest HA-130383 and HA-130384 as potential lead compounds.

## Abstract 2

By the end of 2019, a novel strain of the corona viral family named SARS-CoV-2 emerged in Wuhan, China and started to spread worldwide causing one of the most dangerous lethal pandemics.

## Graphical abstract

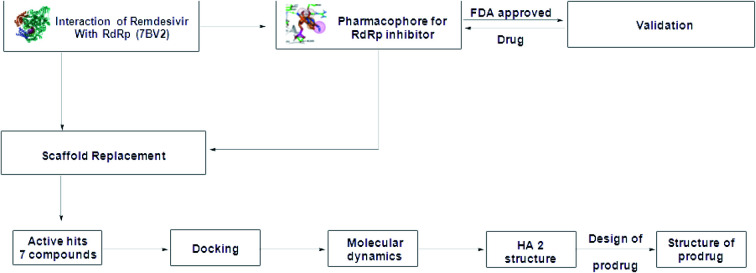

## 1 Introduction

Coronaviruses (CoV) represent a diverse family of enveloped, positive-sense, single stranded RNA viruses that are responsible for many respiratory and enteric diseases in humans.^1^ The most notable highly pathogenic human corona viruses (CoVs) were Severe Acute Respiratory Syndrome corona virus (SARS-CoV) and Middle East Respiratory Syndrome corona virus (MERS-CoV). They were capable of causing a severe fatal respiratory disease.^1^ Both SARS-CoV and MERS-CoV broke out and spread with mortality rates of 10% and 35%, respectively.^1^ By the end of 2019, a novel strain of this family emerged in Wuhan, China and started to spread worldwide causing one of the most dangerous lethal pandemics in years.^2^ Rapidly on the way to discovering a cure, trials were performed to determine one of the viral components that could be targeted and effectively inhibited.^2–6^

Many research groups focused on targeting a virus-specific protein known as RNA-dependent RNA polymerase (RdRp) which is considered as one of 16 non-structural proteins (nsp) that were generated inside SARS-CoV-2.^1,7^ This polymerase (nsp12) is responsible for viral replication.^8^ These research groups utilized various reported inhibitors for virtual screening analyses against this viral target.^2–5,9^ On June 2020 Yin, *et al.* reported and published the crystal structure of the nsp12–nsp7–nsp8 complex bound to the template-primer RNA and triphosphate form of remdesivir active metabolite (RTP).^10^ They noticed that the nsp12, RdRp, possesses minimal activity on its own, and it required the addition of co-factors like nsp7 and nsp8 for full polymerase activity.^10^

Later on, remdesivir, which was an antiviral nucleotide analogue that inhibit the RNA polymerase of the Ebola virus, was investigated as a promising candidate against SARS-CoV-2.^5,11^ Lo, *et al.* suggested the potential mechanism of inhibiting the SARS-CoV-2 RdRp for remdesivir.^12^ According to that, remdesivir acts as a prodrug that is metabolized in cells to yield an active remdesivir triphosphate that could be used by the RdRp as a substrate, leading to the incorporation of remdesivir monophosphate (RMP) into the growing RNA product.^13^ Finally, on October 2020, remdesivir showed promising results against SARS-CoV-2 in cell culture, animals and clinical trials was approved by FDA for the treatment of adult and pediatric patients over 12 years old suffering COVID-19 requiring hospitalization.^14^

**Fig. 1:**
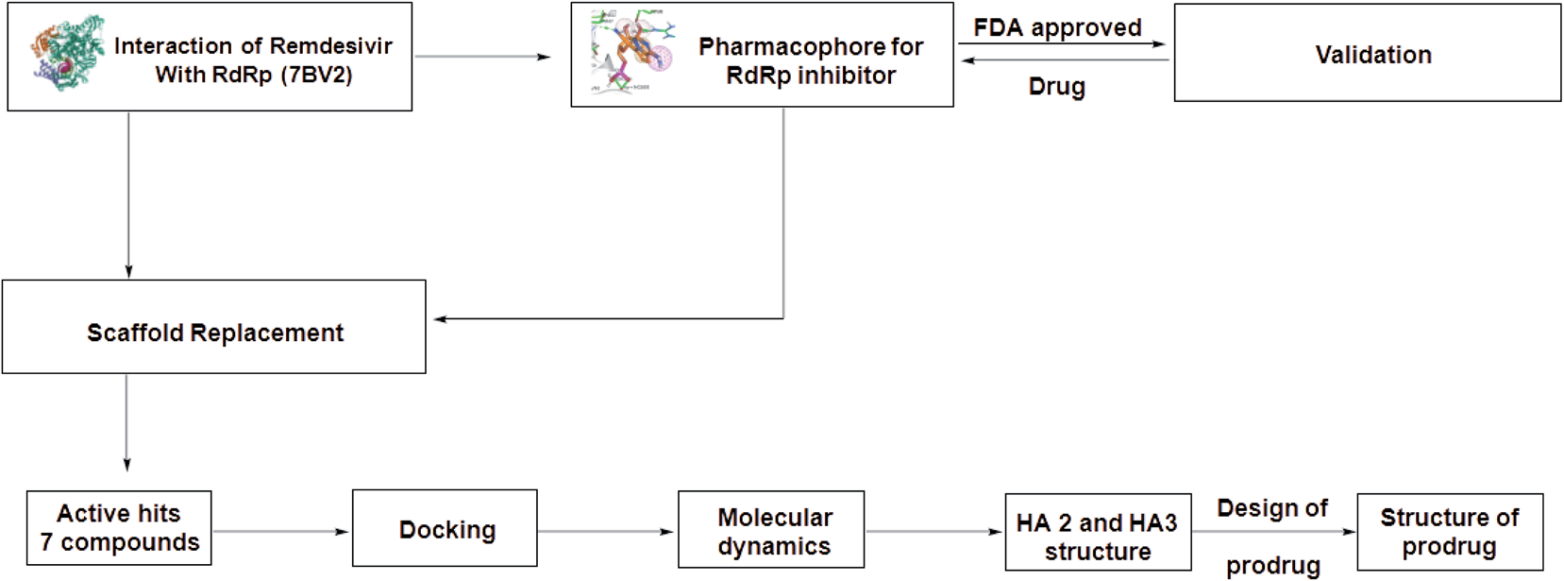
Protocol for the generation of novel SARS-CoV-2 polymerase inhibitor lead.

In spite of the aforementioned data, the use of remdesivir will remain limited by many factors, including the high price of dose regimen related to the low percent of decreasing the mortality rate.^15^ In addition, its I.V. dosage form and its contraindication in both, patients suffering elevation of liver enzymes and those who suffer from renal impairment are important factors that limit its use.^16^ Recently on 19 December 2020, WHO withdrew the use of remdesivir for the approved purpose.^15^ Based on the previously mentioned drawbacks, remdesivir is considered as a proper lead compound and the improvement of remdesivir was an essential need to obtain new analogs with proper activity and minimum drawbacks. This target will be the research interest in this research work following steps demonstrated Fig. 1 as will be discussed later.

## 2 Materials and methods

### 2.1 Protein preparation

SARS-CoV-2 polymerase (PDB 7BV2) was retrieved from protein databank and pyrophosphate, RNA, and water molecules were removed. After removal, the remaining residues were subjected to preparation by quick prep protocol in Molecular operating environment (MOE, 2019.0102) with the use of AMBER forcefield (Amber10: EHT) and R-Field solvation parameter^17^ with standard settings. These settings include protonation of protein and flipping of ASN/GLN/HIS in Protonate3D. Also, refinement for the protein was followed with RMS gradient of 0.1 kcal mol^−1^ A^−1^.

### 2.2 Structure based pharmacophore

The interaction of the co-crystallized metabolite of remdesivir was the guide to select five different features which were 1 Don (radius 1.5), 2 Aro (radius 1) and 2 Acc/Don (radius 1.3). This was achieved from compute/pharmacophore, query editor sequence from the main menu of MOE 2019.0102. The protocol was run according to EHT scheme (extended Huckle theory).^17^ Application of Hückel theory to pharmacophore discovery. CICSJ Bulletin, 33(2), 33 and the strength of the different features was determined as Don > 0.5 and Acc > 0.8. Each feature was assigned from the interactions of co-crystallized ligand with the active site. The radius was adjusted to give a selective pharmacophore through the validation process. The external validation and selectivity were checked by refitting of FDA-US approved database (Antiviral Library from Selleck, Catalog No. L7000) consisting of 302 drug molecules to give only 7 compounds including remdesivir.

### 2.3 Scaffold replacement

Pyrrolotriazine moiety was chosen as the main scaffold to be replaced. This could be done using scaffold replacement panel from compute/fragment/scaffold replacement. Selection of pyrrolotriazine, leads to 9 leaving atoms and 2 exit vectors. The linker database for this process consists of 40 626 fragments varied between aliphatic, aromatic and fused aromatic fragments. The process was constrained by two methods, lead-like descriptor filter and the structure-based pharmacophore. GBVI/WSA dG was the scoring function used in the protocol. Finally, generated database was minimized and clustered according to the score of the scoring function.

### 2.4 Molecular docking

The generated database was prepared to generate bioactive conformers according to the given protocols in MOE 2019.0102. The docking protocol for this process was restricted using the structure-based pharmacophore and refinement of the docking process was performed using rigid receptor. The scoring function of the placement was London dG and for the refinement was GBVI/WSA dG. The whole protocol was validated by re-docking of the co-crystalized remdesivir fragment to give RMSD value 0.2178 Å. The given compounds were clustered by docking score and the best compound was subjected to another docking process with induced fit receptor refinement.

### 2.5 Molecular dynamic simulations

MD simulation was done using GROMACS 2020.3 molecular dynamics package (Abraham *et al.*, 2015 ^18^). Ligand was parameterized using SwissParam^19^ while protein was prepared using all-atom CHARMM36 force field.^20^ Simulations were performed in TIP3P explicit water^21^ and neutralized by Na^+^ ions. Minimization was done using steepest descent algorithm and maximum force was set to less than 1000 kJ mol^−1^ nm^−1^. Systems were initially equilibrated using first constant volume and temperature (NVT ensemble) and then constant pressure and temperature (NPT ensemble) for 1 ns each. Parrinello–Rahman barostat^21^ and V-rescale algorithm^22^ were used to control pressure at 1 atmosphere and temperature at 300 K respectively. The LINear Constraint Solver (LINCS) algorithm^23^ was used for bond's length constraints and particle mesh Ewald (PME) method^24^ was used for long-range electrostatics calculations. For all simulations 2 fs timestep was used. van der Waals cut-off distance (rvdw) was set to 1.2 nm. Initial coordinates were taken from docking pose.

## 3 Results and discussion

### 3.1 Rational of the design

**Fig. 2:**
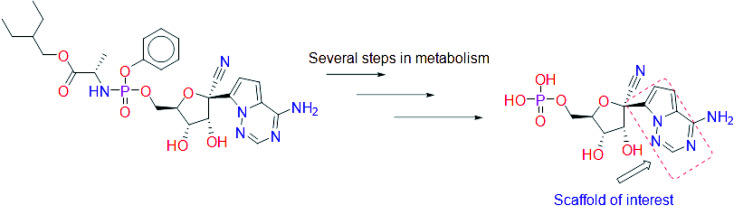
Metabolite of remdesivir and the scaffold of interest that will be replaced to get more active remdesivir analog.

Remdesivir is a prodrug which is activated intracellularly by breakage of side chains attached to phosphate group as shown in Fig. 2. It follows the approach to design some antiviral drugs as protides (prodrugs of nucleotides).^25,26^ This pathway of design was based on the use of nucleoside monophosphate skeleton capped with an aryl group and an amino acid ester (a phosphoramidate).^27^ This process was clear as the metabolic process of remdesivir were extensively studied as reported.^28^ In addition, the metabolite appeared co-crystallized with RdRp of SARS-CoV-2 as shown in the PDB deposit 7BV2.^29^

Due to this outcome, our work will focus on finding remdesivir analogs from a replacement of a scaffold present in the metabolite. Therefore, we designed a workflow for this study depending on scaffold replacement of pyrrolotriazine ring of remdesivir metabolite with respect to retaining the five membered sugar to keep the nucleoside skeleton to follow the protide approach. The side chain of remdesivir is used to ensure the hydrophobicity of the drug to penetrate the cell then it will be removed by metabolic enzymes.

Our protocol will proceed through the following steps; we first create a structure-based pharmacophore which will used to retain the important contacts of remdesivir fragment inside the active site of RdRp. This process will be followed by molecular docking of the generated database form the scaffold replacement process.

### 3.2 Generation of structure-based pharmacophore

**Fig. 3:**
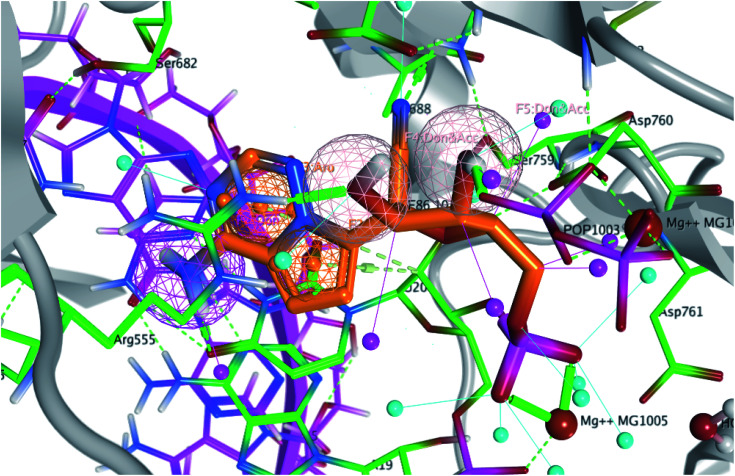
The generated pharmacophore based on the interaction of remdesivir metabolite within the active site of RdRp SARS-CoV-2 (PDB 7BV2). Pharmacophore query consists of 1 Don, 2 Aros and 2 Accs %26 Dons.

The interaction of co-crystallized remdesivir fragment was the guide to choose the annotation points for the pharmacophore query. Remdesivir fragment displayed a hydrogen bond between 5-CN group of the tetrahydrofuran ring with Asn691 and 4-OH from the same ring with Arg555. Different features were selected to retain the interaction (Fig. 3); two hydrogen bond acceptor and donor (Accs & Dons) features to retain the interaction with Arg555. In addition, two aromatic (Aro) features were added to represent the presence of fused system to retain the spatial arrangement of the contacting groups. Finally, a donor (Don) feature was selected due to the 4-amino group on pyrrolotriazine ring as it displayed a hydrogen bond with the dissecting RNA in the 3D structure with U20 as reported.^29^

The generated pharmacophore was validated by pharmacophore search for a decoy test set consists of 302 US-FDA approved drugs (Antiviral Library from Selleck, Catalog No. L7000) for the treatment of SARS-CoV2. Fitting of these compounds to the generated pharmacophore would give 7 compounds including remdesivir as shown in Table 1.

**Table 1:** The output compounds from fitting of the US-FDA approved database in the generated structure-based pharmacophore

Compound name	Structure	RMSDX	Rescoring
Tubercidin	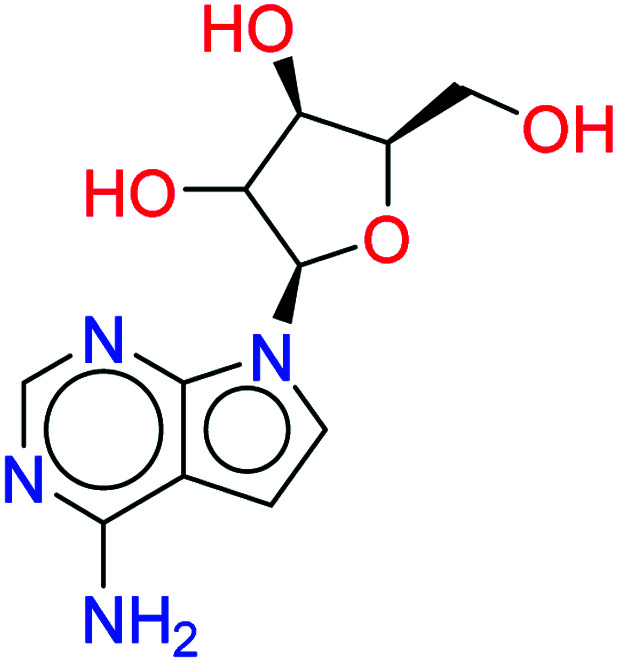	0.7628	6.4849
*N* ^6^-Methyladenosine	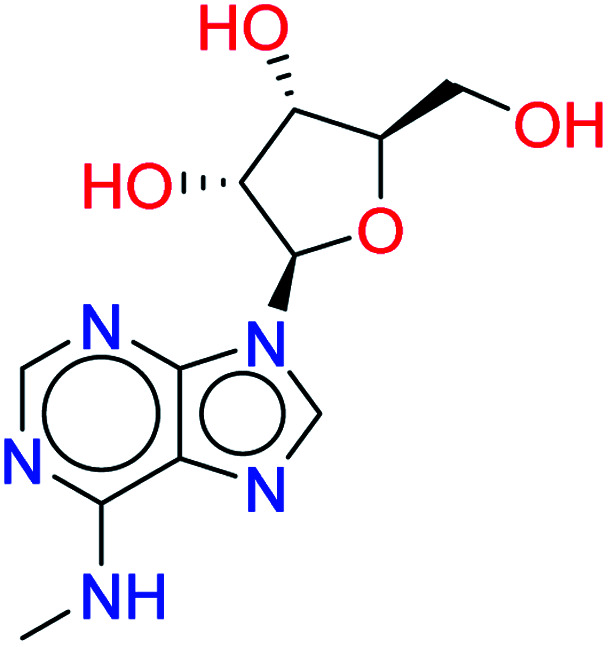	0.7718	4.8280
Vidarabine	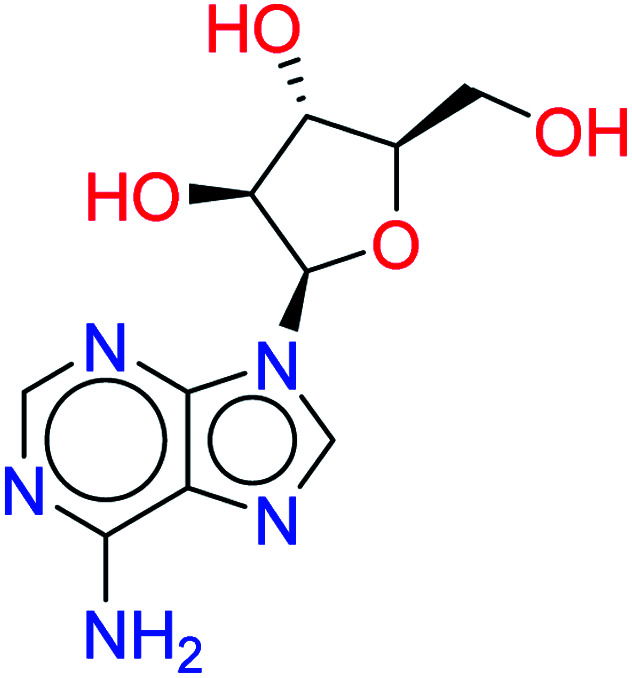	0.7545	4.9939
Rutin	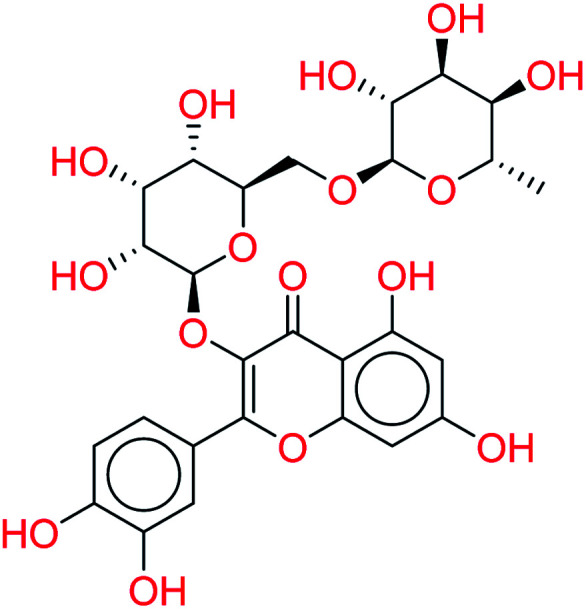	0.7872	3.5647
Orientin	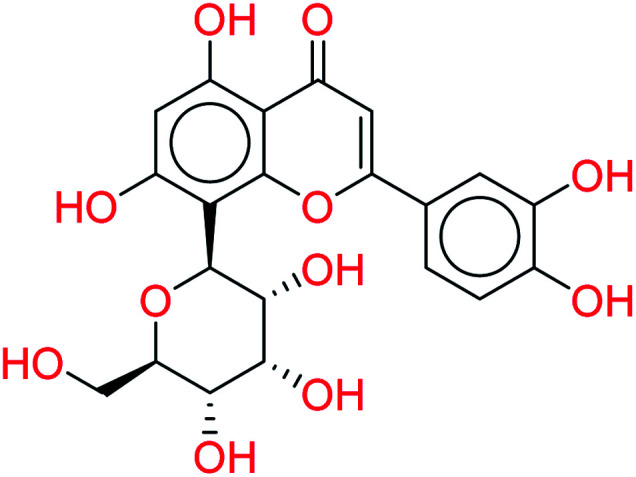	0.9484	5.0431
Shikonin	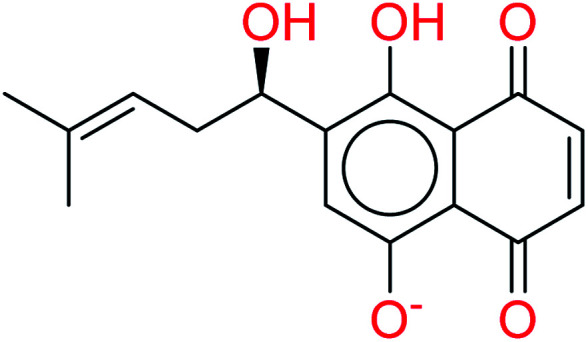	1.0092	3.8107
Remdesivir	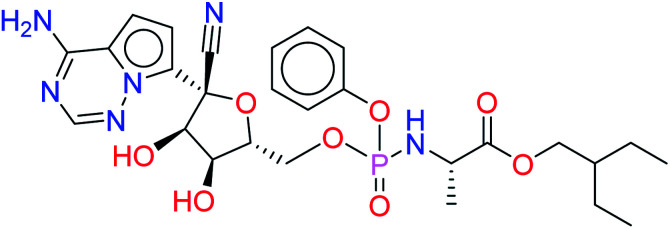	0.2963	5.2112

### 3.3 Scaffold replacement

**Fig. 4:**
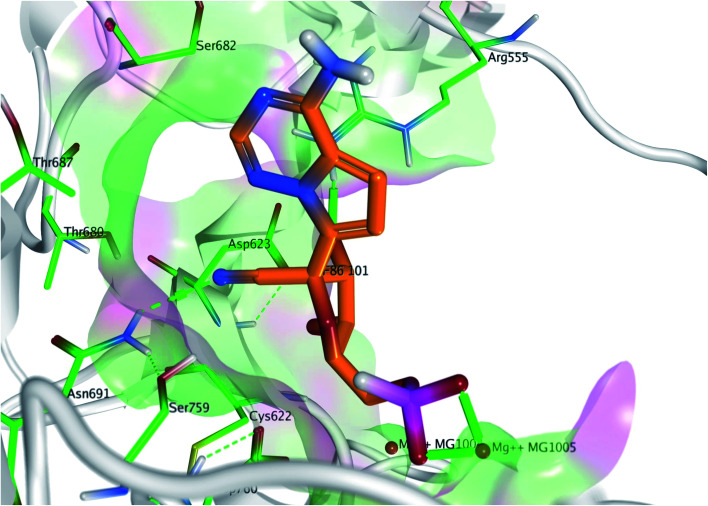
The targeted side area of the pyrrolotriazine fused system of remdesivir within the active site of RdRp SARS-CoV-2 (PDB 7BV2).

Pyrrolotriazine fused system will be the target of the interest in this process. This process will be constrained by the generated structure-based pharmacophore. In this process we will focus on occupying the side area of this fused system as shown in Fig. 4.

The linker database for this step is available in MOE 2019.0102 and consists of 40 626 linkers. This process resulted in a database consisting of 1231 remdesivir metabolite analogs. In the given database, binding free energy was calculated for any given structure according to the scoring function GBVI/WSA dG.^30^ The given metabolites were clustered and arranged according to their scores.

### 3.4 Molecular docking

The given database from scaffold replacement was subjected to molecular docking simulations within the active site of RdRp SARS-CoV-2 (PDB 7BV2). The docking process was restricted by the structure-based pharmacophore to ensure the proper orientation with respect to the active remdesivir fragment. The compounds were clustered according to score and it was observed that all of them with an extension to the replaced fused system and the top five ranked compounds are shown in Table 2. Their new scaffolds are named from the top to the bottom as 4-amino(2-benzyl-1*H*-benzo[*d*]imidazol-1-yl), 4-amino(3-(4-oxobutyl)-1*H*-indol-1-yl), 4-amino-3-((*E*)-2-cyano-3-oxoprop-1-en-1-yl)-1*H*-indol-1-yl, (*Z*)-3-((1*H*-pyrrol-2-yl)methylene)-4-amino-2-oxoindolin-1-yl and finally, 5-amino-3-(1*H*-imidazol-4-yl)naphthalen-1-yl as shown in Table 2.

**Table 2:** The top five ranked compounds after docking process

Metabolite name	Structure	Scoring	RMSD
HA1	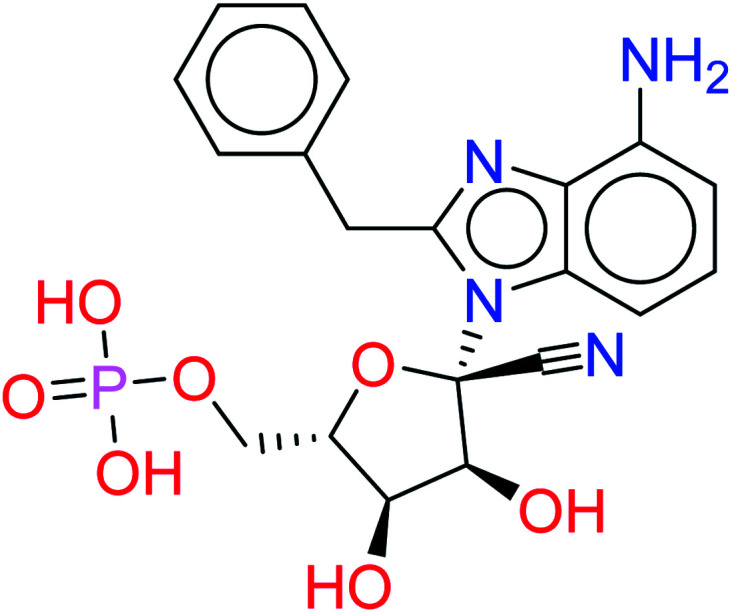	−9.7371	0.0924
HA2	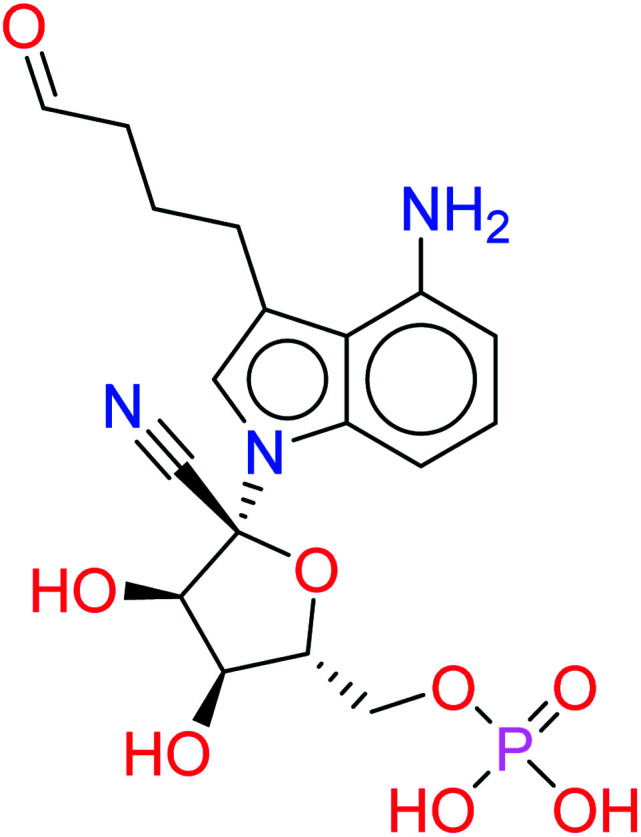	−9.6154	0.0800
HA3	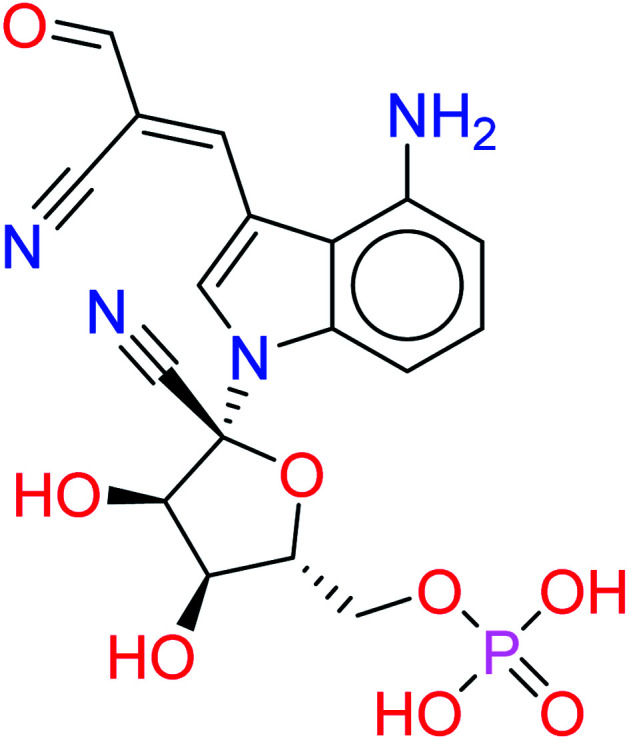	−9.4981	0.0887
HA4	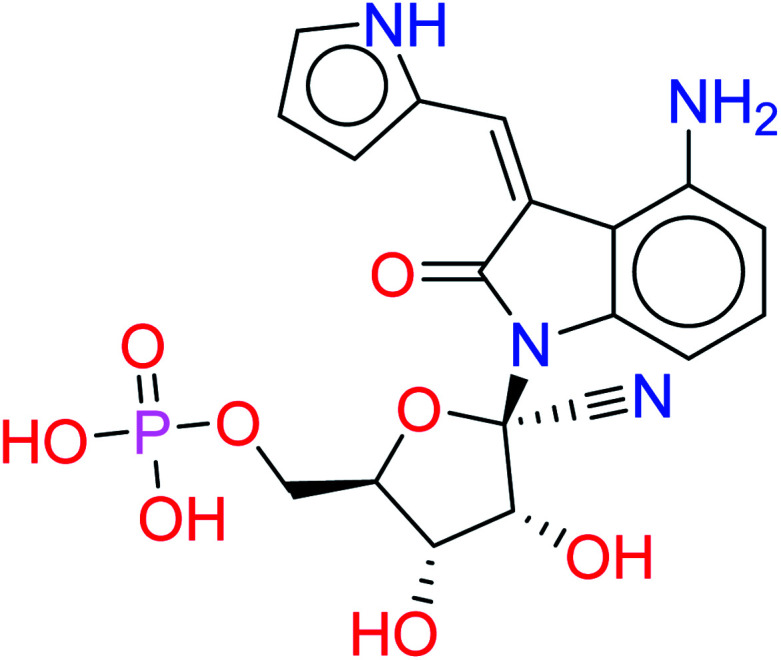	−9.3512	0.0768
HA5	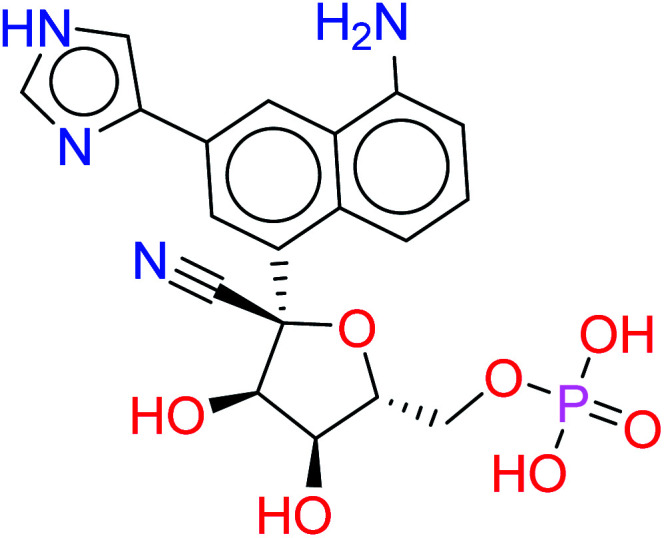	−9.2272	0.1214

**Fig. 5:**
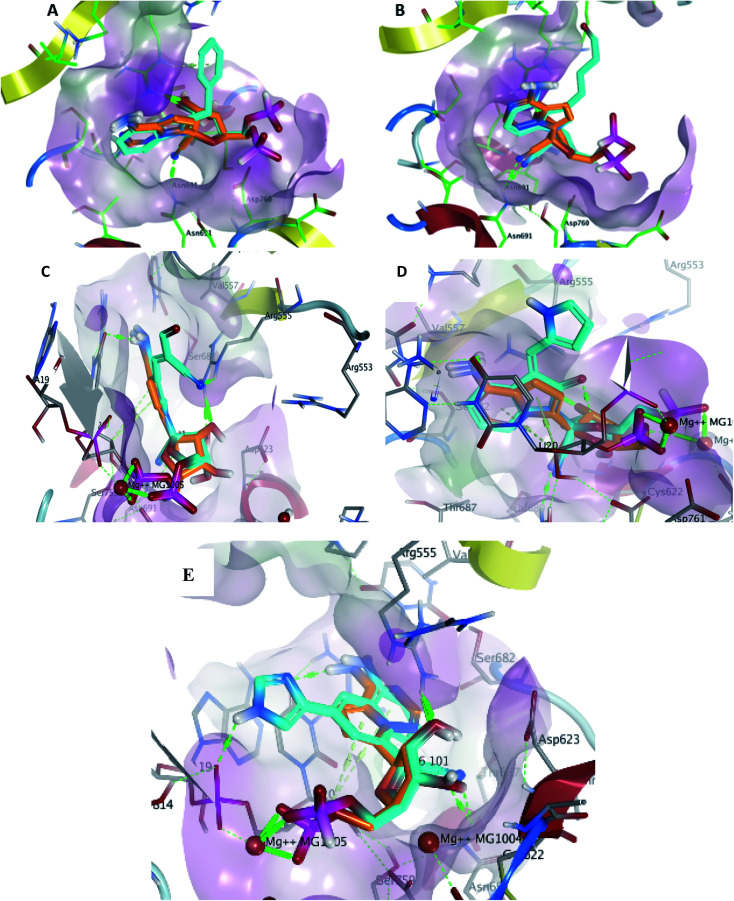
Docking pose of metabolites HA1 (A), HA2 (B), HA3 (C), HA4 (D) and HA5 (E) (cyan) aligned to remdesivir metabolite (orange) within the active site to show the extra contact given by the additional scaffold.

Metabolite HA1 was considered as a good candidate as it was characterized by extra interaction due to the side chain of the fused ring system. It was found that the benzyl ring occupies the vacant pocket to form a π interaction with Arg555 with retaining all other interactions of the co-crystallized metabolite of remdesivir as shown in Fig. 5A.

While metabolite HA2 that was ranked as the second good candidate showed a successful occupying of the vacant pocket nearby Arg555 through its aliphatic side chain in addition to retaining all other interactions of the co-crystallized metabolite of remdesivir as shown in Fig. 5B. All poses of other metabolites were illustrated in Fig. 5.

For more investigation about the given poses, molecular dynamic simulations for the five given docking poses would be taken into considerations in order to assess the stability of these docking poses in the active site.

### 3.5 Molecular dynamic simulations

Molecular dynamics simulations were done to study the stability of the top five compounds in docking study (Table 2) and compare their stability to the co-crystalized ligand (control) as well as the apoprotein. The complexes of these compounds in the active site of SARS-CoV-2 RdRp (PDB 7BV2) were used and their coordinates were obtained from docking poses. The protein used in the molecular dynamics included all the 3 protein chains (nsp12, nsp7 and nsp8). The full nsp12–nsp7–nsp8 complex is known to have the RNA polymerase activity^15^ and hence was included during the MD simulation. After initial minimization and equilibration, the complexes were run for 100 ns production run. Data was collected every 5000 steps (10 ps) of the production run giving 10 000 points to analyze.

**Fig. 6:**
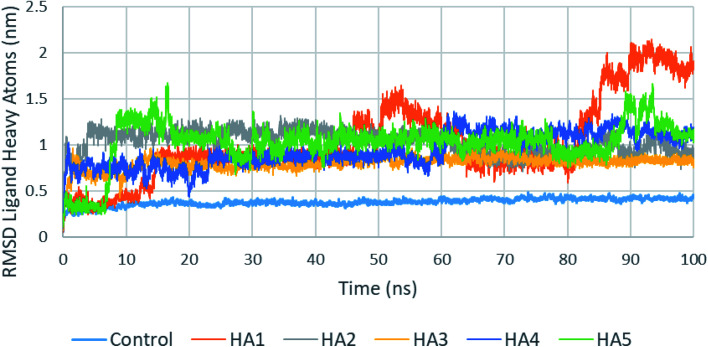
RMSD of tested ligands heavy atoms in the active site of SARS-CoV-2 RdRp during the production run.

One of the most important parameters that could be extracted from the production run trajectories is the root mean square deviation (RMSD) of the ligand heavy atoms. A high fluctuation in the RMSD of ligand heavy atoms indicate that the ligand is not stable in the active site. Fig. 6 shows a plot of ligands heavy atoms RMSD. For examples, the position of the ligand heavy atoms of HA1 was found to be highly fluctuating during the production run during most of the production run especially after 80 ns. An analysis of the studied ligands heavy atoms RMSD is shown in Table 3. As seen in the table, control has a fluctuation of 10.8% of the RMSD average. Among tested compounds (HA1–HA5), HA2 and HA3 showed least fluctuation and hence best stability.

**Table 3:** Ligand heavy atoms RMSD analysis for studies compounds in the active site of SARS-CoV-2 RdRp during the production run

	Control	HA1	HA2	HA3	HA4	HA5
Average RMSD (nm)	0.378	1.030	1.038	0.799	0.940	1.025
Standard deviation	0.041	0.440	0.121	0.071	0.181	0.235
Percent standard deviation (%)	10.80	42.68	11.63	8.83	19.28	22.89

**Fig. 7:**
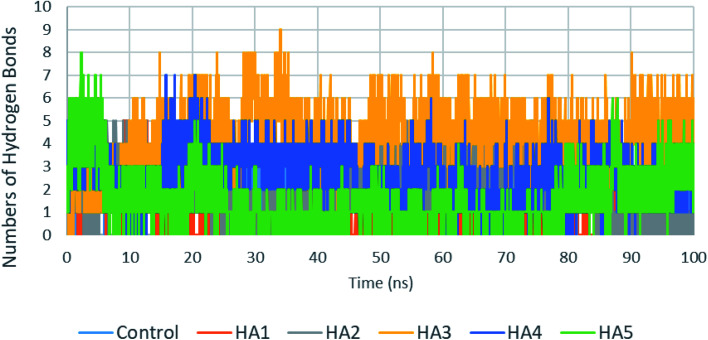
Number of hydrogen bonds formed between tested ligands with RdRp during the production run.

Another important factor that contributes to the stability of ligands in the active site is the number of hydrogen bonds between ligands and proteins. The number of hydrogen bonds between the six studied ligands and target proteins are plotted in Fig. 7. Analysis of these results are shown in Table 4. Compound HA3 has shown the maximum number of hydrogen bonds (4.24 hydrogen bonds) followed by HA4 and HA2 which has shown a number of hydrogen bonds comparable to control (2.07 hydrogen bonds).

**Table 4:** Hydrogen bonds analysis for studies compounds in the active site of SARS-CoV-2 RdRp during the production run

	Control	HA1	HA2	HA3	HA4	HA5
Average HB	2.07	0.66	2.04	4.24	2.59	1.61
Standard deviation	0.65	0.99	1.29	1.29	1.13	1.18

**Fig. 8:**
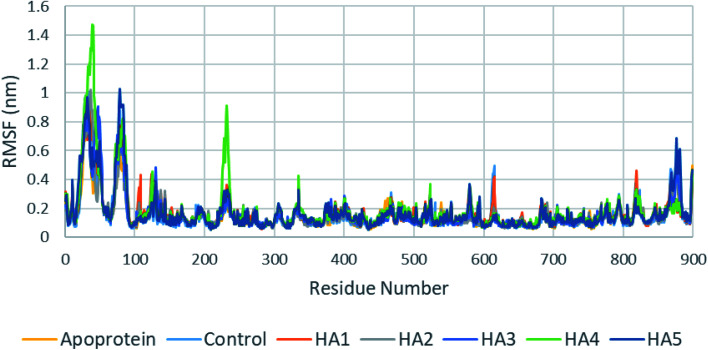
RMSF of SARS-CoV-2 RdRp during production run for apoprotein, control, HA1 and HA2.

In addition, root mean square fluctuation (RMSF) of SARS-CoV-2 RdRp protein residues showed similar results for apoprotein, co-crystalized ligand as well as tested ligands (Fig. 8). It is important to notice that HA4 has shown slightly higher fluctuation in loops around residues 40 and 232 which might indicate movements of these loops to accommodate HA4.

**Fig. 9:**
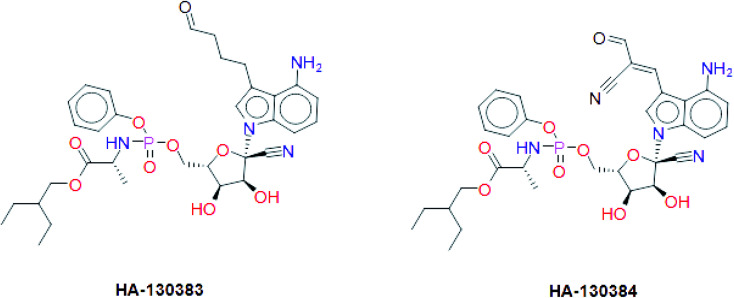
The final suggested remdesivir analogs HA-130383 and HA-130384 of potential inhibitory activity against RdRp of SARS-CoV-2.

These results suggest the higher stability of HA2 and HA3 in the active site of SARS-CoV-2 RdRp during production run. In addition, total interaction energy between ligand and protein was calculated as the total of short-range Lennard-Jones (LJ) and Coulomb interaction. The total interaction energy of HA2 (−278.00 ± 34.43 kJ mol^−1^) and HA3 (−393.13 ± 20.03 kJ mol^−1^) were found significantly better compared to other ligands such as HA1 (−46.14 ± 12.35 kJ mol^−1^) and HA5 (−171.53 ± 22.38 kJ mol^−1^). These results further support the superiority of HA2 and HA3. As a result, the suggested prodrugs in Fig. 9 are potential candidates for further investigations against RdRp of SARS-CoV-2.

## 4 Conclusion

In this study, we tried to obtain a remdesivir analog through studying of the interaction of co-crystallized remdesivir metabolite inside active site of RdRp. The obtained results were the guide to choose the pyrrolotriazine as a scaffold of interest and then annotation points for the pharmacophore query. Creation of a structure-based pharmacophore was performed, and different features were selected to retain the interaction. The generated pharmacophore was validated and a test dataset was fitted to the generated pharmacophore that gave 7 compounds including remdesivir. During this process the linker database supported by MOE 2019.0102 was used and focused on occupying the side area of pyrrolotriazine fused system. The given metabolites were clustered and it was observed that most of compounds with extension of the replaced fused scaffold were having the best score. The given database from scaffold replacement was subjected to molecular docking simulations within the active site of RdRp SARS-CoV-2 (PDB 7BV2). The results showed that metabolite HA2 and HA3 and their corresponding prodrugs HA-130383 and HA-130384 are considered as a good candidate as it has higher stability and lower energy compared to the other studied ligands.

## Conflicts of interest

The authors declare that the research was conducted in the absence of any commercial or financial relationships that could be construed as a potential conflict of interest.

## Author contributions

All authors listed have made a substantial, direct and intellectual contribution to the work, and approved it for publication.

## Supplementary Material
